# Targeting LSD1 in tumor immunotherapy: rationale, challenges and potential

**DOI:** 10.3389/fimmu.2023.1214675

**Published:** 2023-07-07

**Authors:** Lei Bao, Ping Zhu, Yuan Mou, Yinhong Song, Ye Qin

**Affiliations:** ^1^ Hubei Key Laboratory of Tumor Microenvironment and Immunotherapy, China Three Gorges University, Yichang, China; ^2^ College of Basic Medical Science, China Three Gorges University, Yichang, China; ^3^ Department of Nephrology, The First College of Clinical Medical Science, China Three Gorges University, Yichang, China; ^4^ Institute of Infection and Inflammation, China Three Gorges University, Yichang, China

**Keywords:** LSD1, immunotherapy, PD-(L)1, tumor microenvironment, combination therapy

## Abstract

Lysine-specific demethylase 1 (LSD1) is an enzyme that removes lysine methylation marks from nucleosome histone tails and plays an important role in cancer initiation, progression, metastasis, and recurrence. Recent research shows that LSD1 regulates tumor cells and immune cells through multiple upstream and downstream pathways, enabling tumor cells to adapt to the tumor microenvironment (TME). As a potential anti-tumor treatment strategy, immunotherapy has developed rapidly in the past few years. However, most patients have a low response rate to available immune checkpoint inhibitors (ICIs), including anti-PD-(L)1 therapy and CAR-T cell therapy, due to a broad array of immunosuppressive mechanisms. Notably, inhibition of LSD1 turns “cold tumors” into “hot tumors” and subsequently enhances tumor cell sensitivity to ICIs. This review focuses on recent advances in LSD1 and tumor immunity and discusses a potential therapeutic strategy for combining LSD1 inhibition with immunotherapy.

## 1 Introduction

Based on the interactions between the tumor and the immune system, cancer immunotherapy that targets the immune system has revolutionized cancer treatment (1, 2). At present, immunotherapy has developed two mainstream branches: one is immune checkpoint inhibitors represented by PD-(L)1/CTLA4 inhibitors, and the other is adoptive cell therapies represented by chimeric antigen receptor (CAR) T cell therapy, including CAR-NKs (3–6). However, the current reality is that most patients have low response rates to available checkpoint therapies due to a broad array of immunosuppressant mechanisms such as hostile metabolic states, nutritional deprivation, T cell apoptosis triggered, secretion of suppressive cytokines and lack of antigen presentation (1, 3). As a result, the more successful combination medicines are discovered, the more patients will get benefit (3).

Epigenetics is a regulatory process that changes mediating heritable patterns of gene expression without altering the DNA sequence (7). Epigenetic modifications influence immune cells activation, differentiation, and functional fate, and they play critical roles in tumor development, progression, and metastasis (8–10). Histone lysine demethylases (KDMs) are a series of epigenetic enzymes that regulate gene transcription by demethylation of lysine during development and malignant transformation (11). As the first identified KDMs family member, Lysine-specific demethylase 1 (LSD1, also known as KDM1A) also plays an important role in epigenetic regulation (12).

LSD1 was firstly identified by Dr. Shi in 2004, and this discovery also demonstrated that histone methylation is reversible (13). Then LSD1 has gradually become a research hotspot, as it is involved in a variety of physiological and pathological processes, including cancer development, progression, metastases as well as recurrence (14). Of note, although LSD1 is overexpressed in a variety of tumors and has been reported to correlate with overall survival in patients (15–19), it does not seem to be a potent oncogene (20). However, LSD1 regulates gene expression in cancer cells and immune cells, allowing tumor cells to adapt to the tumor microenvironment (TME) (20). Therefore, an in-depth understanding of the role of LSD1 in tumor immunity is critical for developing more effective combination immunotherapeutic targets.

Here, we summarize the regulatory roles and mechanisms of LSD1 on antitumor immunity, including effects on tumor immunogenicity, various immune cells, and cancer-associated fibroblasts (CAFs). Further, we discuss potential innovative therapeutic strategies combining LSD1 inhibitors and multiple immunotherapies to improve the efficacy of mainstream cancer immunotherapies.

## 2 LSD1 and tumor immunity

### 2.1 LSD1 inhibition promotes the tumor immunogenicity

**Figure 1:**
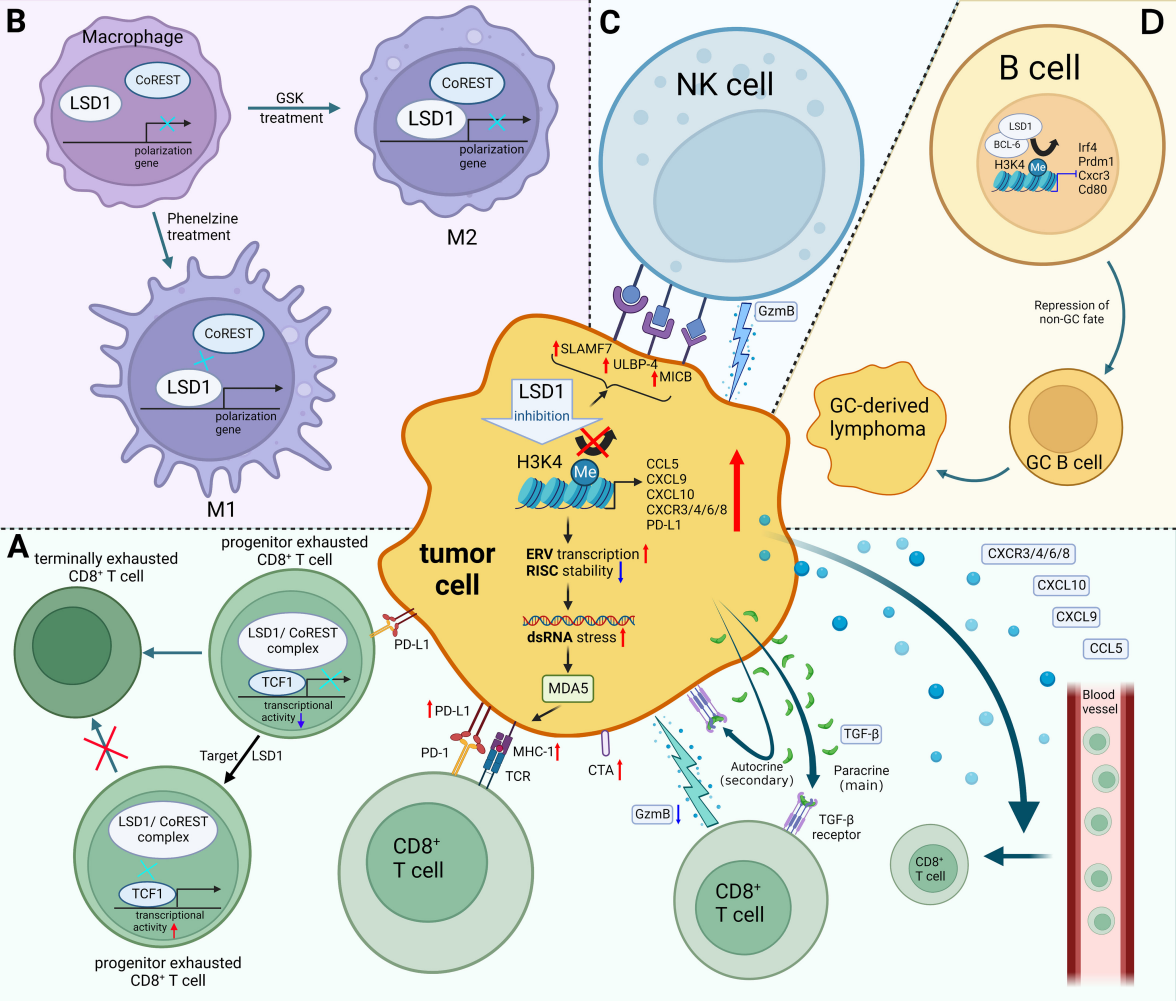
Mechanisms of LSD1 regulating tumor immunity. **(A)** LSD1 inhibition enhances the tumor immunogenicity, promotes CD8^+^ T cell infiltration, and induces TGF-β as well as PD-L1 expression of tumor cells, which provides a potential strategy for enhancing tumor response rates to PD-L1 blockade therapy. There have been few examples in which LSD1 inhibition downregulates PD-L1, e.g. in cervical cancer. Moreover, inhibition of T cell-intrinsic LSD1 sustains T cell invigoration. **(B)** LSD1 inhibition favors M1 macrophage polarization by disrupting the LSD1-CoREST complex. **(C)** LSD1 inhibition confers tumor cells sensitivity to NK cell lysis *via* inducing the expression of ligands on the surface of tumor cells. **(D)** LSD1 induces the progression of GC-derived lymphomas by promoting the differentiation of GC B cells. Red upward arrows indicate upregulation, blue downward arrows indicate downregulation, black arrows indicate transition. Figure created using BioRender.

Recent studies have shown that loss of LSD1 improved tumor immunogenicity, provoking the immune system to fight against tumors (21). Tumor immunogenicity is associated with the expression of tumor-associated antigens (TAA) and tumor-specific antigens (TSA) as well as the ability of tumor antigen presentation (22). However, low or non-immunogenic tumor cells avoid being recognized and killed by immune cells due to weaker antigen expression and presentation capabilities (23), which often associates with poor prognosis (24). Therefore, enhancing the immunogenicity of tumors is a potential immunotherapy strategy. A growing body of evidence suggested that inhibition of LSD1 improves tumor immunogenicity in low or non-immunogenic tumors (**Figure 1A**) (25, 26).

Sheng et al. reported that knocking down LSD1 in tumor cells downregulates RNA-induced silencing complex (RISC) components expression and induces the expression of repetitive elements, including endogenous retroviral elements (ERVs), leading to double-stranded RNA (dsRNA) stress (26). Melanoma differentiation-associated gene 5 (MDA5) senses the accumulation of dsRNA, which is similar to a viral infection (viral mimicry), this leads to activation of innate antiviral pathways, resulting in the production of type I and type III interferon (IFN) as well as the processing and presentation of antigens (23, 26, 27). Meanwhile, knockout of LSD1 promoted MHC-1 expression on the surface of tumor cells (26). Likewise, Zhou et al. also proved that inhibition of LSD1 could activate the expression of genes associated with antigen processing and presentation through the ERV-dsRNA-IFN pathway (28).

Cancer testis antigens (CTAs) promote immune system recognition and killing of tumor cells by increasing tumor immunogenicity (25). The reactivation of CTAs in tumors is considered an ideal immunotherapy target because they are not expressed in most antigen-presenting cells from normal tissues (29). It is worth noting that inhibition of LSD1 could upregulate the expression of a range of representative CTAs, which enhanced tumor immunogenicity (25).

Collectively, these studies suggested that blockading LSD1 promotes tumor immunogenicity in multiple tumor models and provides a new therapeutic strategy for immunotherapy of low-immunogenic or non-immunogenic tumors (25, 26).

### 2.2 LSD1 regulates CD8^+^ T cell

#### 2.2.1 LSD1 inhibition promotes CD8^+^ T cell infiltration

Lymphocytes that infiltrate the tumor are called tumor-infiltrating lymphocytes (TILs) (30). According to TILs abundance, tumors have been divided into “cold tumors” versus “hot tumors” (31, 32). Currently, a pathway that can turn “cold tumors” into “hot tumors” is urgently needed, due to the poor clinical response by “cold tumors” (23, 33). “Cold tumors” are characterized by a lack of T lymphocyte infiltration, whereas “hot tumors” are typified by the infiltration of CD8^+^ cytotoxic T cells (31, 34). In particular, infiltration of CD8^+^ T cells is known to be associated with favorable prognosis (35). Hence, it is critical to explore ways to activate CD8^+^ T cells infiltration into the TME. A growing number of studies had shown that LSD1 blockade increases CD8^+^ T cell infiltration in the tumor tissue and promotes anti-tumor immunity (**Figure 1A**) (26, 36–40).

A recent study demonstrated that LSD1 ablation does not increase the expression of Granzyme-B (a cytotoxic factor) and Ki-67 (a proliferation marker), but significantly promotes the infiltration of T effector cells into the melanoma cells and then restrains tumor growth (26). Besides, Ji et al. observed the increasing proportion of CD8^+^ T cells and the ratio of CD8^+^ T cells to Tregs (CD8/Treg) in TME of triple-negative breast cancer (TNBC) when treating with an innovative hydrogel-loaded LSD1 inhibitor GSK-LSD1 (36). Likewise, LSD1 inhibitor SP-2509 promoted CD8^+^ T cell infiltration in head and neck squamous cell (HNSCC) and oral squamous cell carcinoma (OSCC) cells (37, 38). Interestingly, suppression of LSD1 simultaneously promoted the infiltration of CD8^+^, CD4^+^, CD4^+^CD8^+^ double positive T cells and CD56^+^ NKT cell infiltration in small cell carcinoma of the ovary hypercalcemic type (SCCOHT) (39).

Mechanistically, LSD1 blockade increases the enrichment of H3K4me2 at proximal elements or core regions of the transcription start site of CD8^+^ T cell-attracting chemokine promoters, which induces the expression of CD8^+^ T cell-attracting chemokines (CCL5, CXCL9, CXCL10), thereby promoting the infiltration of CD8^+^ T cell into tumor tissues and exerting tumor-killing effects (40). Similarly, LSD1 expression is inversely proportional to T cell chemokine gene expressions, such as CXCR3, CXCR4, CXCR6, CXCR8, CCL5, CXCL9, and CXCL10 in HNSCC (37). Notably, other chemokines such as CCL2, CCL3 or CCL4 are recognized to have tumor promoting effects (41). Those chemokines’ expression is insignificantly regulated by LSD1 expression (40).

Taken together, LSD1 inhibition increases CD8^+^T cell infiltration by inducing tumor cells to secrete CD8^+^ T cell-attracting chemokine. This may turn “cold tumors” (immunotherapy-insensitive) into “hot tumors” (immunotherapy-sensitive).

#### 2.2.2 LSD1 inhibition sustains T cell invigoration

Programmed death-ligand 1 (PD-L1) expressed in tumors interacts with programmed death receptor 1 (PD-1), resulting in prolonged stimulation of T cell receptor (TCR) by cognate antigens, inducing CD8^+^ T cells to differentiate into exhausted CD8^+^ T cells (Tex cells) (42). Under persistent antigen stimulation, progenitor Tex cells differentiate into terminally exhausted T cells (43). Current evidence suggested that the progenitor Tex cells had better cytokine-producing and proliferation capacity, and could maintain self-renewal while continuously producing more cytotoxic differentiated cells (44). T-cell factor 1 (TCF-1) was identified as a key transcription factor during progenitor Tex cells differentiation (45).

Mechanistically, LSD1/nuclear REST corepressor 1 (CoREST) complex interacts with the long isoform of TCF-1 in progenitor Tex cells and inhibits the transcriptional activity of TCF-1, thereby promoting terminal differentiation of progenitor Tex cells (46). It could be reversed by suppression of T cell-intrinsic LSD1, which increases the persistence of progenitor Tex cells and provides a continuous source of proliferative conversion into numerically greater terminally Tex cells with tumoricidal cytotoxicity (**Figure 1A**) (46).

#### 2.2.3 LSD1 suppression induces TGF-β expression of tumor cells

TGF-β plays a crucial role in immune homeostasis and tolerance, which is secreted by cancer cells and several other cells present in the TME (47). It was upregulated in LSD1-knockout tumor cells and antagonized the antitumor effects of LSD1 inhibition-induced CD8^+^ T cell infiltration (48). Currently, TGF-β has three well-known mechanisms accounting for tumor immune escape, including repressing the cytotoxicity of CD8^+^ T cells (49), promoting the conversion of CD4^+^CD25^-^ T cells to T(reg) cells (50), and blocking T cells infiltration (51, 52). Nevertheless, the latter two mechanisms did not appear to be decisive for antagonizing the antitumor effects induced by LSD1 inhibition. For example, fluctuations of TGF-β levels did not lead to significant alternation in Treg cell frequency in B16 (48) and EMT6 (51) tumors. In addition, CD8^+^ T cell infiltration was not further increased in tumor cells knocked out of both LSD1 and TGF-β comparing to tumor cells knocked out of LSD1 alone, which suggested that TGF-β increased by LSD1 blockade did not significantly block CD8^+^ T cell infiltration (48). This is somewhat expected since IFN pathway activation is more important than TGF-β pathway activation for CD8^+^ T cell infiltration induced by LSD1 inhibition (26, 48).

In particular, TGF-β has two opposing effects in tumors according to its different targets’ cells (48). Primarily, paracrine TGF-β attenuates the cytotoxicity and the tumor-killing ability of CD8^+^ T cells through its action on αβ T cells, thereby reducing the percentage of GzmB^+^ CD8^+^ TILs. Secondarily, autocrine TGF-β inhibits tumor growth by acting directly on tumor cells to partially inhibit cell cycle progression and promote tumor cell apoptosis. Overall, the tumor-promoting effect of paracrine TGF-β is stronger than the tumor-inhibitory effect of autocrine, that TGF-β induced by LSD1 inhibition helps tumors escape from host immune responses by repressing the anti-tumor activity of CD8^+^ cytotoxic T cells (**Figure 1A**) (48). Hence, inhibiting or blocking the paracrine effect of TGF-β is one of the potential strategies to enhance the tumor-killing effect of LSD1 inhibitors (48).

### 2.3 LSD1 inhibition favors M1 macrophage polarization

Macrophages have different phenotypes and functions in different microenvironments, and they are divided into two categories according to their function: M1 macrophages (classically activated macrophages) and M2 macrophages (alternatively activated macrophages) (53, 54). Currently, increasing studies had demonstrated that LSD1 could regulate macrophages polarization (55–60).

In non-tumor tissues, activation of the LPS/TLR4/NFκB/PARP1-LSD1/SOD2 signaling pathway regulates the resistance of M1 macrophages to hydrogen peroxide (55). The mechanism mentioned was that LSD1 represses SOD2 transcription by enriching in the SOD2 gene promoter region and increasing H3K4 demethylation. Thus, LSD1 inhibition can prevent hydrogen peroxide-induced oxidative stress damage to M1 macrophages by promoting SOD2 transcription (55). Notably, Sobczak et al. observed that LSD1 suppression promoted catalase expression during M1 polarization, which in turn inhibited the expression of pro-inflammatory cytokines and M1-related surface markers (such as CD14, TNF-α, COX2, IL1-β, IFNAR, and TLR2), which suggested that LSD1 inhibition can limit the macrophage M1 specialization in the non-tumor tissues (56).

In the TME, M1 macrophages exert anti-tumor effects, while M2 macrophages promote tumor proliferation, metastasis, and angiogenesis (61). Therefore, inducing the polarization of M1 macrophages in the TME provides a potential therapeutic strategy for treatment of tumors (62). Of note, Boulding et al. reported that LSD1 blockade promotes the M1 macrophage polarization and infiltration (57). They observed the increased expression of CCR7 and CD38 (M1 markers) and the decreased expression of CD206 and EGR2 (M2 markers) in the MDA-MB-231 tumor tissues following treatment with LSD1 inhibitor phenelzine (57). Moreover, significantly higher infiltration of M1 macrophages after the combination therapy of phenelzine and nab-paclitaxel was observed, which implied that LSD1 blockade could serve as a potential epigenetic adjuvant therapy strategy (57). Interestingly, Phenelzine, an LSD1 inhibitor targeting the flavin adenine dinucleotide (FAD) and CoREST binding domains, increased the transcription and expression of M1-associated genes by disrupting the LSD1-CoREST complex. In contrast, GSK2879552, an LSD1 inhibitor targeting the FAD domain, failed to polarize macrophages to the M1 phenotype (**Figure 1**) (58). These evidences emphasized the importance of targeting the LSD1-CoREST complex to reprogram macrophages toward M1 phenotype for therapeutic benefit.

Current studies showed that inhibition of LSD1 not only inhibits the proliferation and migration of mixed lineage leukemia (MLL) rearranged leukemia cells, but also increases the proportion of macrophages in peripheral blood and spleen (59, 60). The cells expressing high levels of CD11b and CD14, surface-specific markers of differentiated macrophages/monocytes, were significantly increased after LSD1 inhibition (59). Similarly, the percentages expressing CD11b or CD14 were also significantly upregulated following treatment with a structurally new LSD1 inhibitor (spirooxindole-based FY-56) in MLL-rearranged leukemia cells (60). These results might be attributed to differentiation of stem-like leukemia cells into more mature macrophage-like cells caused by LSD1inhibition (59, 60).

### 2.4 LSD1 inhibition confers tumor cells sensitivity to NK cell

Natural killer (NK) cells, as an important member of the immune tumor microenvironment, limit the growth and spread of cancer cells (63). It is well known that NK cells are activated upon detection of abnormal signals of malignant transformation. Once activated, NK cells secrete pro-inflammatory cytokines and lyse target cells *via* the perforin/granzyme pathway (63).

Current research had shown that catalytic LSD1 inhibitors could induce the expression of ligands on the surface of tumor cells that could activate NK cells (**Figure 1C**) (64, 65). Bailey et al. reported that irreversible catalytic LSD1 inhibitors (RN-1, tranylcypromine and GSK-LSD1) could induce NK cells to kill tumor cells (65). Mechanistically, LSD1 inhibition could increase the expression of innate immune receptors (SLAMF7, MICB, and ULBP-4) on the surface of tumor cells in diffuse pontine glioma (DIPG). These receptors act as self-ligating or as ligands for natural killer group 2 member D (NKG2D) to activates NK cells, sensitizing tumor to NK cell lysis (65). Similarly, Liu et al. reported that LSD1 inhibition upregulated the expression of innate immune receptors in acute myeloid leukemia (AML) cells with low expression of CCAAT/enhancer-binding protein α (C/EBPα) (64). They further demonstrated that the expression of C/EBPα was upregulated after treatment with LSD1 inhibitor tranylcypromine which was enriched at the enhancer region of the *ULBP2/5/6* genes, and subsequently induced the ULBP2/5/6 which were ligands for NK cell receptors and activate NK cells by binding to NKG2D. In this way, catalytic LSD1 inhibitors confer sensitivity of tumor cells to NK-mediated lysis (64).

Notably, the two classes of inhibitors targeting different domains of LSD1 have different biological effects on NK cells (20). In contrast to catalytic inhibitors, the reversible scaffolding LSD1 inhibitors (SP-2577 and SP-2509) inhibits NK cells metabolism and lysis capacity (66). Mechanistically, scaffold LSD1 inhibitors downregulates NK cell ligand expression and attenuates NK cell toxicity, whereas glutathione supplementation abolishes these effects and rescues NK cell lysis capacity (66). Thus, glutathione supplementation might relieve the inhibition of NK cell activity when treated with LSD inhibitors.

### 2.5 LSD1 regulates B cells involved in tumor progression

There is a close relationship between tumor-infiltrating B cells and tumors. An analysis of 69 available studies found that B cell infiltration is associated with a positive patient prognosis in 19 tumors, while less than 10% of the studies indicated the opposite phenomenon (67). And it was also reported that LSD1 is required for B cell proliferation and differentiation (68, 69).

In recent years, studies have shown that different infiltration patterns or different directions of B cells induced by TME, therefore, B cells play two opposite roles of anti-tumor and tumor-promoting (67, 70). Interestingly, LSD1 acts as a tumor promoter or suppressor in some different tumors, due to the regulation of B cell differentiation by LSD1 (71–73). On the one hand, LSD1, a germline predisposition gene for multiple myeloma, inhibits multiple myeloma development by regulating abnormal plasma cells (PC) (72). On the other hand, LSD1 induces the progression of germinal center (GC)-derived lymphomas by promoting the differentiation of GC B cells (**Figure 1D**) (73). Mechanistically, LSD1 and the transcriptional repressor BCL6 forms a complex that subsequently represses the expression of genes involved in GC exit, terminal differentiation as well as proliferation, thereby inducing GC B-cell differentiation and promoting the progression of GC-derived lymphomas (73). Notably, conditional deletion of LSD1 inhibited GC proliferation, while catalytic LSD1 inhibitors have little effect on GC proliferation and lymphoma progression (73). Therefore, the development of novel inhibitors that target non-catalytic LSD1–protein interactions might become an attractive therapeutic intervention for GC-derived lymphomas (71).

### 2.6 The connection between LSD1 and CAFs

CAFs are abundant in the TME and closely related to cancer progression. CAFs affects tumor cells and other stromal cells through cell-to-cell contacts, release a variety of regulatory factors, synthesize and remodel the extracellular matrix, thereby impacting the cancer progression (74). Current research suggests that there is a connection between LSD1 and CAFs (57, 75).

CAFs induced LSD1 deacetylation and maintain LSD1 stability by activating Notch3 signaling, resulting in the promotion of cancer stem-like cell (CSC) self-renewal and tumor growth (75). Another study identified that CAFs increased in the TME following mono-chemotherapy with nab-paclitaxel, whereas CAFs decreased following LSD1 inhibitor administration alone or in combination with chemotherapy in the MDA-MB-231 mouse xenografts (57). This research demonstrated that suppression of LSD1 could effectively reduce the CAFs burden (57). However, the specific subtypes of CAFs that affected by LSD1 remain to be further investigated.

## 3 LSD1 in immunotherapy

### 3.1 LSD1 inhibitor combined with PD-1/PD-L1 blockade

PD-L1 is commonly found on the surface of tumor cells, which inhibits CD8^+^ T cell cytotoxicity and leads to CD8^+^ T cell exhaustion by binding to PD-1 on the surface of T cells, thereby mediating immune escape of tumor cells (44, 76). Therefore, PD-1/PD-L1 blockade promotes anti-tumor immunity and kill tumor cells (77). Some cancer patients who initially responded to anti-PD-(L)1 therapy eventually develop drug resistance and tumor progression after long-term treatment, though PD-1/PD-L1 therapy elicits more potent antitumor activity in some patients (78, 79). It should be noted that in most cancer patients, the PD-1/PD-L1 pathway is not the only speed-limiting factor of anti-tumor immunity, so blocking the PD-1/PD-L1 pathway alone is not sufficient to elicit effective antitumor immune response (79). On the one hand, negative factors such as other immune checkpoints, immunosuppressive immune cells or cytokines, cancer-associated adipocytes, abnormal angiogenesis, hyperactive CAFs contribute to tumor immune tolerance (80–85). Removing these negative factors might overcome drug resistance. On the other hand, positive factors such as immune supporting cytokines, immunogenic cancer cell death, and professional antigen-presenting cells promote immune clearance (86). Strengthening these positive factors might reshape “cold tumors” into “hot tumors”, thereby increasing the response rate to PD-1/PD-L1 blockade therapy (86).

It has been validated that epigenetic modulators might be an appropriate partner with PD-1/PD-L1 blockade to achieve superior antitumor efficacies and long-term cancer control (79). LSD1 blockade, as a novel strategy for epigenetic regulation, enhances antitumor effects through multiple sides as discussed previously. On the tumor cell intrinsic side, LSD1 suppression promotes antigen processing and presentation and induces ligand expression. In immune cells, LSD1 suppression regulates the development, differentiation, cytotoxicity, and cytokine production of T cell, and involves in the regulation of macrophages, NK cells, and CAFs in TME, thereby turning “cold tumors” into “hot tumors”.

Existing studies had shown that LSD1 was involved in the regulation of immune checkpoints on the surface of tumor cells. For example, knockdown of LSD1 directly downregulated the expression of PD-L1 and CD47 in cervical cancer through increasing the enrichment of H3K4me2 at promoters of PD-L1 and CD47 (87). Besides, the LSD1/wild-type p53/miR-34a signaling axis indirectly regulated the expression of CD47/PD-L1 by targeting the 3’ untranslated region (3’ UTR) of CD47/PD-L1. Further studies reported that combination therapy with PD-(L)1/CD47 blockade and LSD1 inhibition significantly inhibited tumor growth compared with the single-agent treatment group (87). However, LSD1 blockade upregulated PD-L1 expression in most tumors, including melanoma (26), SWI/SNF-deficient ovarian cancer (39), HNSCC (37) and OSCC (38). Likewise, the expression of PD-L1 was proved to be increased by LSD1 inhibitor HCI-2509 in a dose-dependent manner in MDA-MB-231 cells and mouse TNBC cell line models 4T1 and EMT6 (40). H3K4me2 occupancy at a distant region upstream of the TSS site of PD-L1 promoters was enhanced after LSD1 inhibition. Meanwhile, the enrichment of H3K4me2 at proximal elements or core regions of transcription start site at promoters of PD-L1 was increased (**Figure 1A**) (40). This explains why inhibition of LSD1 induces PD-L1 in a variety of tumors.

Given the dramatic effect of LSD1 inhibition in enhancing tumor immunogenicity and promoting T cell infiltration, combination with LSD1 suppression and PD-(L)1 blockade may have potential therapeutic value (26). Several observations support this hypothesis. For example, LSD1-knockout B16 mice showed a slow increase in tumor volume and significantly prolonged survival after PD-1 blockade (26). Another study points out that tumor grew significantly more slowly in BALB/c mice bearing orthotopic EMT6 tumors following combination therapy with HCI-2509 and PD-1 blockade. Likewise, combination treatment inhibited tumor growth and lung metastasis in 4T1 tumor-bearing BALB/c mice, compared with single-agent treatment (40). These had also been demonstrated in HNSCC (37) and OSCC (38). These studies suggest that the combination of LSD1 inhibition and PD-(L)1 blockade is a potential strategy for anti-tumor immunotherapy.

In addition to regulating the expression of PD-L1 on the surface of the cell membrane as discussed previously, LSD1 deletion had been shown to reduce the expression of exosomal PD-L1 (88). PD-L1 is released from tumor cells and exists in extracellular forms, including soluble PD-L1 and exosomal PD-L1 (89). Existing studies suggest that exosomal PD-L1 played an important role in tumor immune escape, promoting tumor development by inhibiting cytokine production and promoting T cell apoptosis (90, 91). Correspondingly, reducing the content of exosomal PD-L1 might enhance the sensitivity of tumor patients to anti-PD-L1/PD-1 therapy (89). Shen et al. reported that LSD1 deletion could reduce PD-L1 accumulation in exosomes and inhibit PD-L1 transport to other cancer cells *via* exosomes, thereby enhancing the activity of T cells and restoring the ability of T cells to kill tumor cells in TME, thus overcoming immunosuppression (88).

Nevertheless, the limitations of combination therapies of LSD1 inhibition and PD-(L)1 blockade remain to be resolved. For example, LSD1 suppression-induced TGF-β acted on αβ T cells and reduces the toxicity of CD8 ^+^ T cells. This limited the anti-tumor immune response of the dual-combination therapy to some extent (48). Therefore, the triple-combination of PD-1/TGF-β blockade and LSD1 inhibition had been shown to effectively inhibit tumor cell growth through increasing the cytotoxicity and infiltration of CD8^+^ T cells. Triple therapy overcomes the limitations of dual therapy and provides a new treatment strategy for low-immunogenicity tumors (48).

It is worth noting that tumor cells are not the only target of LSD1 inhibition therapy. The progenitor Tex cells is reported as the key determinant of effective responses to anti-PD1 therapy (92, 93). Inhibition of T cell-intrinsic LSD1 disrupted the interaction of the LSD1/CoREST complex with TCF-1 in Tex progenitor cells, which in turn induced TCF-1 transcriptional activity, thereby inhibiting the terminal differentiation of Tex progenitor cells (46). This expanded the pool size of progenitor Tex cells, leading to durable and effective responses to anti-PD1 therapy (46).Taken together, blockade of T cell-intrinsic LSD1 provides another promising target for epigenetic modulation in cancer immunotherapy.

Collectively, combination therapy with PD-(L)1 blockade and LSD1 inhibition reduce tumor growth more effectively. These results suggest that inhibition of LSD1 may be an effective adjunct to immunotherapy, broadening potential therapeutic strategies for low-immunogenic or non-immunogenic tumors. Such a phase I and phase II clinical trial combination with LSD1 inhibitor and anti-PD-1 is currently recruiting lung small cell carcinoma patients (NCT05191797). In addition, based on the combination therapy of inhibiting LSD1 and blocking PD-(L)1, further inhibition of tumor growth-promoting cytokines (e.g.TGF-β) induced by LSD1 inhibition could potentially improve the effectiveness of combination therapy for poorly immunogenic tumors.

### 3.2 LSD1 inhibitor combined with CAR-T therapy

In recent years, research on CAR-T cell therapy has grown exponentially due to its tremendous clinical success in lymphoma and leukemia patients (94). CAR-T cell therapy enables T cells to bind tumor cell surface antigens through antigen-binding domains (usually a single chain variable fragments (scFv)), mediating MHC-unrestricted tumor cell killing (95). CAR-T cell mainly kills tumor cells through the granzyme perforin pathway, but the Fas/FasL pathway has been shown to be closely related to the killing ability of CAR-T cell on tumor cells (96). However, overcoming drug resistance of treating solid tumors and further improving the efficacy of treating leukemia and lymphoma are still the most challenging issues in CAR-T cell therapy (97, 98). Hence, the discovery of promising new targets and the innovative design of CAR-T cells are crucial (94).

Recent studies have shown that inhibition or knockout of LSD1 can indirectly or directly enhance the ability of CAR-T cells to kill tumor cells (99, 100). Sulejmani et al. showed that inhibiting LSD1 in tumor cells promoted TP53-mediated transcriptional activation of genes, which leads to increased expression of Fas on the tumor cell surface, allowing FasL on CAR T cells to bind to Fas on the surface of tumor cells lacking antigen expression, thereby lysing and killing tumor cells (99). It should be noted that the above results are based on *in vitro* experiments, and it is necessary to further study the toxicity and effectiveness of this strategy *in vivo* through animal experiments (99). Unlike Sulejmani O et al. who targeted LSD1 in tumor cells, Zhang J et al. suggested that targeted knockdown of LSD1 in anti-CD19 CAR-T cells have stronger anti-tumor effect (100). *In vitro* experiments showed that the knockdown of LSD1 promoted anti-CD19 CAR-T cells to secrete IFN-γ, TNF-α, and IL-2 and enhanced their cytotoxic and cytolytic activities. *In vivo* experiments showed that LSD1-knockdown anti-CD19 CAR-T cells exhibited stronger IFN-γ secretion capacity and better expansion rate. This suggested that LSD1 downregulation may contribute to the long-term antitumor activity of anti-CD19 CAR-T cells (100).

These studies suggested that LSD1 may become a promising adjuvant strategy for CAR-T cell therapy and provide new ideas for the innovative design of CAR-T cells.

## 4 Conclusions

As a histone lysine demethylase, LSD1 regulates chromatin domains that are activated or repressed by histone demethylation, which modulates the expression of immune cell-related genes, thereby affecting the tumor immune response in the TME. LSD1 blockade, as a novel strategy for epigenetic regulation, enhances antitumor effects through multiple sides. On the tumor cell intrinsic side, LSD1 suppression promotes antigen processing and presentation. Some important ligands expression also can be induced by LSD1 suppression. In immune cells, LSD1 suppression regulates the development, differentiation, cytotoxicity, and cytokine production of T cells, and is involved in the regulation of macrophages, NK cells, and CAFs in TME, thereby turning “cold tumors” into “hot tumors”. In brief, inhibition of LSD1 can inhibit tumor immune escape and effectively kill tumor cells through multiple mechanisms. Furthermore, inhibition of LSD1 suppresses the progression of GC-derived lymphomas by inhibiting the differentiation of GC B cells. However, whether LSD1 inhibition can suppress tumorigenesis and tumor development by inducing immune cells to differentiate into subtypes remains to be studied. Overall, the extensive effects of inhibiting LSD1 on tumor immunity need to be fully explored.

Although anti-PD-(L)1 antibody therapy and CAR-T therapy are currently the most popular immunotherapy strategies, it is undeniable that immunotherapy is less than ideal for a variety of cancers. Current researches focus on the efficacy of LSD1 inhibition combined with anti-PD-(L)1 antibody therapy and CAR-T therapy. More evidences are needed to determine whether LSD1 blockade is suitable as a potential combination strategy for more immunotherapies such as CTLA-4 inhibitors or CAR-NK therapy. Altogether, targeting LSD1 may offer an exciting avenue to improve the efficacy of immunotherapy.

## Author contributions

YQ and YS contributed to conception and design of the study. LB and PZ completed the review of literature and wrote the first draft of the manuscript. YM contributed to the graphic visualization. All authors contributed to the article and approved the submitted version.
